# Travel distances, socioeconomic characteristics, and health disparities in nonurgent and frequent use of Hospital Emergency Departments in South Carolina: a population-based observational study

**DOI:** 10.1186/s12913-015-0864-6

**Published:** 2015-05-16

**Authors:** Brian K. Chen, Xi Cheng, Kevin Bennett, James Hibbert

**Affiliations:** Arnold School of Public Health, University of South Carolina, 915 Greene Street Suite 354, Columbia, South Carolina 29208 USA; School of Medicine, University of South Carolina, Columbia, South Carolina USA

**Keywords:** Nonurgent use, Emergency department, Primary care access, Health disparities

## Abstract

**Background:**

Nonurgent use of hospital emergency departments (ED) is a controversial topic. It is thought to increase healthcare costs and reduce quality, but is also considered a symptom of unequal access to health care. In this article, we investigate whether convenience (as proxied by travel distances to the hospital ED and to the closest federally qualified health center) is associated with nonurgent ED use, and whether evidence of health disparities exist in the way vulnerable populations use the hospital ED for medical care in South Carolina.

**Methods:**

Our data includes 6,592,501 ED visits in South Carolina between 2005 and 2010 from the South Carolina Budget Control Board and Office of Research and Statistics. All ED visits by South Carolina residents with unmasked variables and nonmissing urgency measures, or approximately 76 % of all ED visits, are used in the analysis. We perform multivariable linear regressions to estimate correlations between (1) travel distances and observable sociodemographic characteristics and (2) measures of nonurgent ED use or frequent nonurgent ED use, as defined by the New York University ED Algorithm.

**Results:**

Patients with commercial private insurance, self-pay patients, and patients with other payment sources have lower measures of nonurgent ED use the further away the ED facility is from the patients’ home address. Vulnerable populations, particularly African American and Medicaid patients, have higher measures of nonurgent ED scores, and are more frequent users of the ED for both nonurgent and urgent reasons in South Carolina. At the same time, African Americans visit the hospital ED for medical conditions with higher primary care-preventable scores.

**Conclusions:**

Contrary to popular belief, convenient access (in terms of travel distances) to hospital ED is correlated with less-urgent ED use among privately insured patients and self-pay patients in South Carolina, but not publicly insured patients. Unequal access to primary care appears to exist, as suggested by African American patients’ use of the hospital ED for primary care-treatable conditions while experiencing more frequent and more severe primary care-preventable conditions.

**Electronic supplementary material:**

The online version of this article (doi:10.1186/s12913-015-0864-6) contains supplementary material, which is available to authorized users.

## Background

Visiting hospital emergency departments (ED) for nonurgent medical conditions is a controversial topic. In the United States, high-end estimates consider approximately 56 % of more than 120 million annual visits to the hospital ED to be potentially avoidable, costing an estimated $38 billion in additional healthcare expenditures [1]. Conservative estimates place nonurgent ED use at 8 % of annual ED visits, by patients of all payer and age groups, and contrary to popular belief, even by patients with a usual place of care [2].

In this article, we separate use of the hospital ED into four broad categories: care received for medical conditions not requiring treatment within 12 h, care that could have been safely and effectively provided in a primary care setting, care for conditions that could have been prevented with timely and appropriate primary care, or care requiring unavoidable emergency treatment [3]. We consider the first two types of ED use to be nonurgent. Although opinions differ, some believe that such use of the ED may contribute to negative consequences such as ED overcrowding [4], increased lengths of stay [5-7], early departures of patients requiring care [8], and ED care rationing [9, 10]. Moreover, the episodic nature of ED care makes it inappropriate as a “medical home” for vulnerable patients requiring coordination of care. However, many consider nonurgent use of the hospital ED as a symptom of barriers to primary care access for vulnerable populations [11-14].

Despite substantial controversy surrounding nonurgent ED use, there is surprisingly little population-based research on the observable factors associated with nonurgent (as opposed to frequent) ED use. Many of the existing studies drew their samples from a single institution, had samples smaller than 500 individuals, or applied only bivariate or descriptive statistics [15]. To supplement and extend the results of these studies to a population level, we used all available in-state ED visits from South Carolina to assess correlations between observable factors (patient socioeconomic characteristics as well as travel distances) and measures of ED visit urgency. Our overarching goal is to uncover, on a population level, whether travel distances and patient characteristics are associated with nonurgent ED use.

## Methods

### Data

We obtained our ED data from the South Carolina Office of Research and Statistics (ORS) all-payer database from 2005 to 2010. In addition, we merged South Carolina’s ORS all-payer inpatient data with the ED observations using unique encrypted patient identification numbers (IDs) to identify those ED visits with a subsequent inpatient admission within 30 days after the index ED visit. This indicator of subsequent hospitalization serves as a test of construct validity for our primary measures of nonurgent ED use.

To investigate patient choices of treatment facilities near their residence, we restricted our sample to South Carolina residents based on their residential zip code, and dropped observations with missing variables or apparently incorrect admission dates, which resulted in a total of 6,592,501 ED observations in our analytical sample (76 % of the full sample).

### Outcomes

Our primary outcome variables are measures of nonurgent use based on the New York University Emergency Department Algorithm (NYU ED Algorithm) [3]. This algorithm was developed by a panel of ED and primary care physicians who analyzed the records of approximately 6,000 ED visits in New York area hospitals in 1988 and assigned a value between 0 % and 100 % that represents the percentage of cases with a particular ICD9 code to be: (1) nonemergent (NE: immediate care not required within 12 h); (2) primary care treatable (PCT: care is required within 12 h, but could have been treated safely in an outpatient setting); (3) primary care preventable (PCP: emergency care is required, but could potentially have been avoided with timely and appropriate ambulatory care); and (4) emergent/unavoidable (EUA: emergency care required, and could not have been avoided with ambulatory care).

At the individual-encounter level, these percentage values, expressed as numbers between 0 and 1, can be conceptualized as the probability that a given diagnosis belongs in any one of the four ED classifications enumerated in the previous paragraph. For example, a primary diagnosis of 786.50 (chest pain, unspecified) has a probability of 0 for being NE, 0.324 for PCT, 0 for PCP, and 0.676 for EUA.

Given that our primary outcome variables are bounded between 0 and 1, we took the natural log of these probability values to reduce positive skewness and to satisfy a key requirement for ordinary least-squares regressions. This regression specification is also useful to estimate approximate percentage changes of the dependent variables associated with unit changes in the explanatory variables. In addition, we created a categorical variable that is equal to 1 if either the NE or the PCT variable is greater than 0. This variable, or NEPCT, is an indicator that denotes a nonzero probability that patients could have chosen not to go to the ED for their medical condition (based on the principal diagnosis in the ED). In a separate specification that studies the association between frequent ED users based on estimated medical urgency, we aggregated, for each patient, the numbers of ED visits respectively with NE, PCT, PCP, and EUA scores of 0.75 or greater between 2005 and 2010.

To test the construct validity of an algorithm developed using data from New York some 20 years ago for analysis in our South Carolina population, we also used an indicator variable equal to 1 if a hospitalization occurred within 30 days after an index ED visit, an indicator variable equal to 1 if patients were referred for inpatient admission during the ED visit, and the natural log of total charges during the ED visit.

#### Independent variables

Covariates included all demographic and socioeconomic variables available for analysis in the all-payer database. We separated age into four categorical variables: aged 1 year and under, 1–17 years, 18–34 years, 35–64 years, and 65 years and over. Patient sex was indicated as female = 1. We coded patient race as African American, Asian, Native American, Hispanic, or Other. Expected source of payment was represented by the indicator variables: Self Pay, Medicare, Medicaid, Health Maintenance Organization (HMO), and Other Payer. In addition, we created separate day-of-the-week categorical variables for the day of the ED visit, from Monday through Saturday. The omitted categories of patients in our specification are White, male gender, aged 65 years and older, commercial health insurance plan, and ED visit on a Sunday.

We obtained the list of all federally qualified health centers and look-alikes (collectively, FQHCs), their service addresses, and their dates of operation from the Health Resource and Service Administration’s data warehouse [16]. From this dataset, we constructed an “FQHC distance” variable as the shortest road distance from the patient’s exact residence address to the closest FQHC in operation during the quarter of the patient’s ED encounter. Likewise, we calculated an “ED distance” variable to represent the shortest road distance between the patient’s residence address and the ED facility visited. To analyze whether travel distances to the nearest FQHC or to the ED facility had any impact on the NYU ED Algorithm scores of ED use based on the patient’s expected source of payment, we interacted the two distance variables with the full set of payer categorical payer variables.

This study was exempted by the University of South Carolina Institutional Review Board because it used only deidentified secondary data. All analyses were conducted using Stata software (v. 13.0).

### Analysis

We employed a multivariable regression model with quarterly and hospital fixed effects. Our methodology is based on the econometric analysis of panel data, a type of longitudinal data in which outcomes associated with a given entity are measured repeatedly over time. We used an econometric fixed-effects model (rather than the fixed-effects model commonly used in biostatistics), where each unique quarter of the year and each unique hospital ID (except one quarter and one hospital ID) is given a separate indicator variable. The attractiveness of this model is that it allows each hospital to serve as its own control; therefore, any unmeasured confounding that remains constant over time is zeroed out. For example, the coefficient on “Medicaid” is estimated by comparing the ED urgency scores of Medicaid patients with the scores of commercial insurance patients (the reference group) at the *same ED* in the *same quarter*. This empirical strategy is considered in the econometric literature to be a consistent estimator in the presence of time-invariant unmeasured confounding [17]. Furthermore, the model assumes that the observed values of the explanatory variables are nonrandom and differ across entities. In our study context, this assumption appears reasonable because it is unlikely that patients of different race, sex, insurance type and status, and travel distances are distributed randomly across hospital EDs in South Carolina.

For the specification with NEPCT as the dependent variable, we chose the linear probability model because a specification using log-transformed dependent variables would drop all NEPCT observations with a 0 value, leaving only observations with a value of 1. Although alternative models such as the fractional probit model exist for specifications with dichotomous outcome variables, the calculation of standard errors for such models with fixed effects is computationally intensive for a dataset of over 6.5 million observations. Indeed, it has been shown that the linear probability model yields coefficient estimates and significance test results substantially similar to those of a fixed-effects fractional probit model [18].

We used ordinary least-squares regression to estimate the correlations between (a) the numbers of ED visits with over 75 % probability of being NE, PCT, PCP, and EUA, respectively, and (b) sociodemographic, payer, and distance covariates. For these regressions, we no longer had a panel data structure because we collapsed the 6 years of data to count the number of ED visits by unique patient ID, but we continued to take the natural log of the dependent variable to reduce positive skewness.

## Results

### Summary statistics

Our final sample includes 6,592,501 ED visits after applying the exclusion criteria (Table 1). Between 2005 and 2010, ED patients aged 1 year and under, 1–17 years, 18–34 years, 35–64 years, and 65 years and over represent 5.4 %, 17.9 %, 30.7 %, 35.8 % and 10.2 %, respectively, of the ED visits in South Carolina. Women represent 57.2 % of the study sample. White, African American, Hispanic, Asian, Native American, and individuals identified as “Other” comprise 53.6 %, 41.9 %, 2.3 %, 0.3 %, 0.2 %, and 1.7 %, respectively, of ED visits. These figures are striking because the 2007 United States census shows that 67.2 % and 29.5 % of South Carolinians were White and African American, respectively [19]. For the expected source of payment, the study sample consists of 26.9 % self-pay patients, 14.7 % Medicare, 23.2 % Medicaid, 27.1 % commercial insurance, 3.4 % HMO, and 4.7 % patients with other sources. Average NE, PCT, PCP, and EUA probability scores are 25.2 %, 25.8 %, 7.0 %, and 12.7 %, respectively.

**Table 1:** General characteristics of study subjects

Variables	Mean	Std. Dev.	Min	Max
	6,592,501			
Age				
0 – 1	0.054			
1 – 17	0.179			
18 – 34	0.307			
35 – 65	0.358			
>65	0.102			
Gender				
Female	0.572			
Race/Ethnicity				
White	0.536			
African American	0.419			
Hispanic	0.023			
Asian	0.003			
Native American	0.002			
Other	0.017			
Payer type				
Self-pay	0.269			
Medicare	0.147			
Medicaid	0.232			
Commercial insurance	0.271			
HMO	0.034			
Others	0.047			
Day of the week				
Monday	0.151			
Tuesday	0.152			
Wednesday	0.144			
Thursday	0.140			
Friday	0.136			
Saturday	0.134			
Sunday	0.143			
Distances				
Distance to hospital	11.685	17.155	0	579
Distance to FQHC	10.610	10.083	0	60
NYU ED Scores				
Nonemergent	0.252	0.336	0	1
Primary Care Treatable	0.258	0.283	0	1
Primary Care Preventable	0.070	0.184	0	1
Emergent	0.127	0.231	0	1

**Fig. 1:**
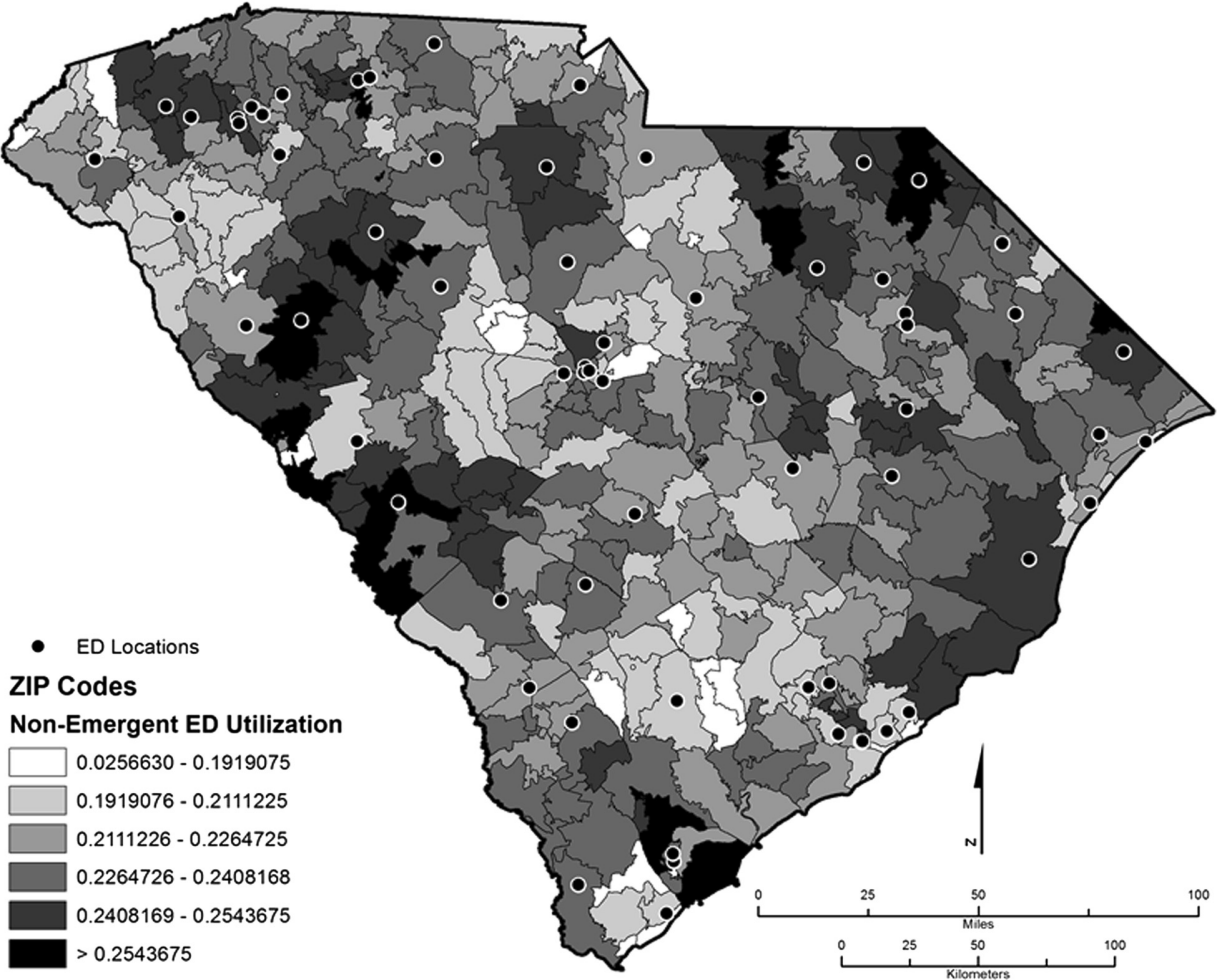
Distribution of nonemergent ED utilization

**Fig. 2:**
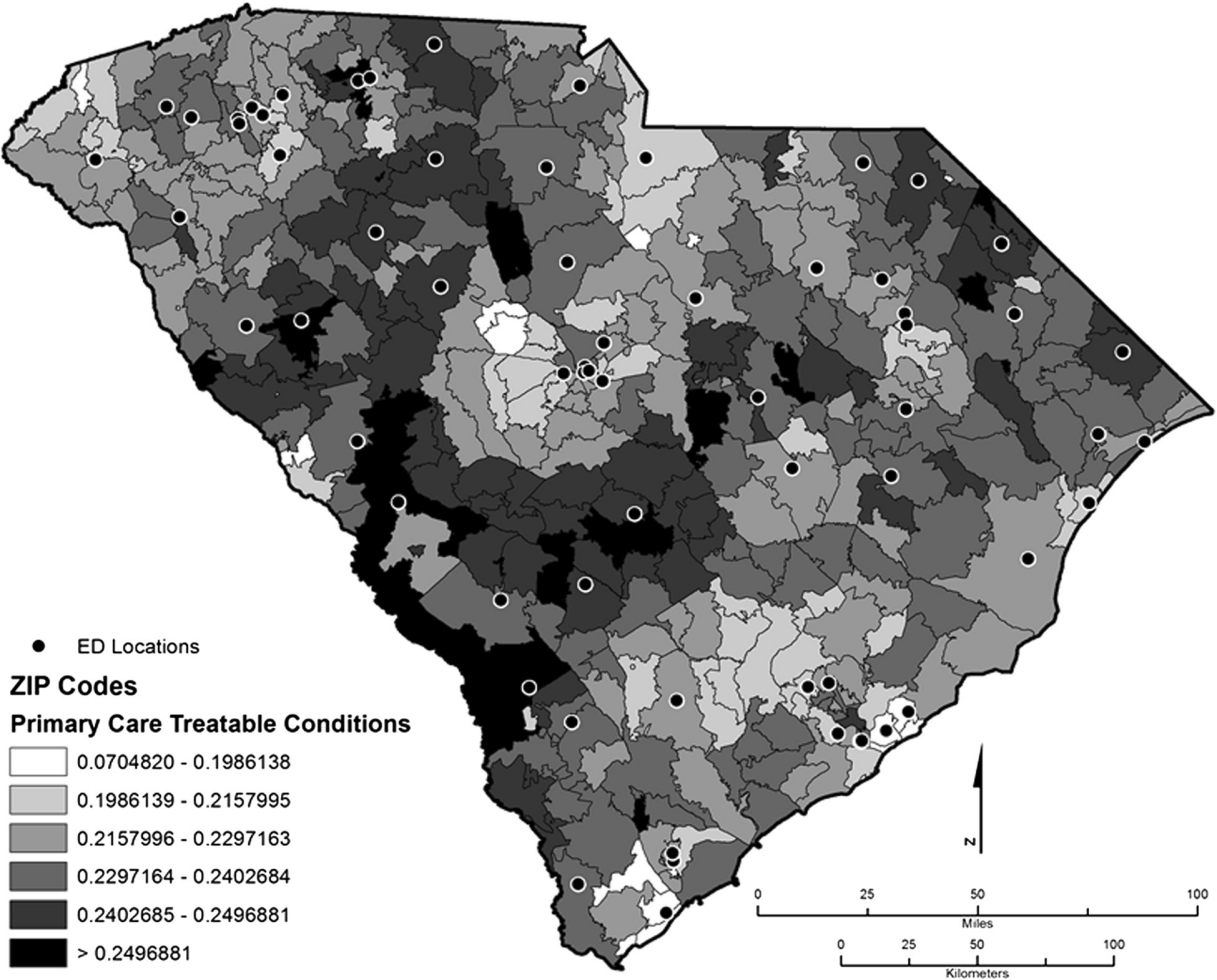
Distribution of primary care treatable ED utilization

In Figs. 1 and 2, we present the distribution of NE and PCT ED use geographically. The most important observation is that while some overlaps occur, the distribution of NE and PCT ED use patterns are not identical. The ED locations in both figures show that apart from South Carolina’s three major urban population centers (Greenville, Columbia, and Charleston) with multiple EDs, there is generally one ED distributed throughout the state’s rural areas. However, the intersection of Orangeburg, Dorchester, and Berkeley counties in the southeastern corridor of the state lacks a centrally located ED. Not surprisingly, these zip codes had lower scores for NE and PCT ED use.

### Travel distances and NYU ED use scores

For ease of interpretation, we present our results in terms of probability points (i.e., with decimals moved two places to the right). Our analysis on travel distances to the ED facility shows that the further the ED is from the patient’s residence, the lower the measures of nonurgent use (NEPCT) for patients with nonpublic or no insurance coverage – commercial insurance (−0.0432, Standard Error (SE) 0.00682), self-pay (−0.0699, SE 0.00708), HMO (−0.0392, SE 0.0819), and patients with other payment source (−0.103, SE 0.0191). For commercial insurance patients, each additional mile traveled to the ED is associated with a reduction in the NE score (−0.0118, SE 0.00350) (Table 2).

**Table 2:** Observable correlates of Nonurgent ED utilization

	(1)	(2)	(3)	(4)	(5)
Variables	NEPCT	Log NE	Log PCT	Log PCP	Log EUA
Age					
≤1	18.6**	−29.7**	37.5**	−94.3**	−52.8**
	(0.541)	(0.849)	(1.25)	(2.08)	(2.58)
1 – 17	−2.21**	−9.06**	8.47**	−71.2**	−45.3**
	(0.473)	(0.590)	(0.866)	(1.67)	(1.66)
18 – 34	4.29**	7.10**	1.45**	−38.7**	−19.8**
	(0.485)	(0.373)	(0.436)	(1.02)	(1.63)
35 – 64	4.25**	6.82**	−4.93**	−11.1**	−15.0**
	(0.325)	(0.305)	(0.397)	(0.778)	(0.988)
Gender					
Female	9.62**	0.405*	2.15**	−7.52**	−5.39**
	(0.142)	(0.170)	(0.382)	(0.447)	(1.19)
Race/Ethnicity					
African American	8.68**	1.07**	−0.453	2.24**	−9.31**
	(0.266)	(0.115)	(0.344)	(0.635)	(0.544)
Asian	2.62**	−2.12**	2.39**	0.0803	1.32
	(0.735)	(0.665)	(0.890)	(1.77)	(2.05)
Native American	7.03**	−0.773	1.85	−2.58	−6.62**
	(1.50)	(1.04)	(1.04)	(3.92)	(1.42)
Hispanic	8.24**	−3.16**	3.76**	−8.76**	0.190
	(0.474)	(0.297)	(0.738)	(1.17)	(1.00)
Other race	4.80**	−1.87**	2.44**	−6.78**	1.80
	(0.476)	(0.387)	(0.490)	(1.10)	(1.35)
Expected source of payment					
Self-pay	1.04	0.715*	2.39**	−1.83*	−16.4**
	(0.611)	(0.331)	(0.389)	(0.763)	(1.19)
Medicare	2.99**	−0.982**	0.809	15.2**	−0.628
	(0.446)	(0.334)	(0.507)	(0.844)	(1.50)
Medicaid	6.17**	2.00**	2.22**	0.255	−6.75**
	(0.585)	(0.407)	(0.447)	(0.927)	(1.34)
HMO	1.02*	−1.74**	−0.242	−1.07	4.72**
	(0.386)	(0.361)	(0.598)	(1.36)	(.0.634)
Other payer	−8.26**	0.894	−0.705	7.22**	−4.93
	(1.74)	(0.511)	(0.702)	(1.65)	(2.68)
Travel distance to FQHC by payment source					
Commercial insurance × FQHC distance	0.0386	−0.0150	0.0102	0.0477	0.172**
	(0.0224)	(0.0158)	(0.0183)	(0.0363)	(0.0512)
Self-pay × FQHC distance	0.0209	0.0106	0.0328	0.00681	0.113*
	(0.0288)	(0.0159)	(0.0241)	(0.0368)	(0.0475)
Medicare × FQHC distance	0.0636**	0.00615	0.0520**	0.0047	0.0570
	(0.0177)	(0.0194)	(0.0183)	(0.0520)	(0.0542)
Medicaid × FQHC distance	0.0268	−0.00777	0.0451	0.0556	0.0447
	(0.0164)	(0.0159)	(0.0280)	(0.0397)	(0.0336)
HMO × FQHC distance	0.0596	0.0365*	0.00984	0.125*	0.0421
	(0.0328)	(0.0176)	(0.0322)	(0.0525)	(0.0548)
Other payer × FQHC distance	−0.106	0.0258	−0.0160	−0.122*	0.0952
	(0.0813)	(0.0216)	(0.0334)	(0.0605)	(0.107)
Travel distance to ED by payment source					
Commercial insurance × ED distance	−0.0432**	−0.0118**	−0.0128	0.0184	0.0691**
	(0.00682)	(0.00350)	(0.00817)	(0.0139)	(0.0108)
Self-pay × ED distance	−0.0699**	0.0219**	−0.0220**	0.0299*	0.0649**
	(0.00703)	(0.00485)	(0.00713)	(0.0123)	(0.0129)
Medicare × ED distance	0.00524	−0.00612	0.00664	−0.0251	0.0422*
	(0.00859)	(0.00811)	(0.0113)	(0.0232)	(0.0177)
Medicaid × ED distance	−0.00974	−0.00335	−0.0357**	0.0888**	0.106**
	(0.00809)	(0.00686)	(0.0130)	(0.0242)	(0.0190)
HMO × ED distance	−0.0392**	−0.00919	−0.0231	0.0323	0.0613**
	(0.00819)	(0.00648)	(0.0150)	(0.0328)	(0.0202)
Other payer × ED distance	−0.103**	0.00470	0.0163	−0.0707	0.0487
	(0.0191)	(0.0107)	(0.0147)	(0.0364)	(0.0416)
Constant	90.2**	−44.2	−51.7**	−65.8**	−55.5**
	(0.701)	(7485)	(1.35)	(1.61)	(2.48)
Observations	6,592,501	2,619,932	4,184,293	1,507,037	2,556,219
R-squared	0.041	0.066	0.018	0.125	0.042
Number of hospitals	64	64	64	64	64

For legibility, all decimal points on the coefficients have been moved two places to the right and can be interpreted as probability percentage points. NEPCT is an indicator variable that is equal to 1 if either NE or PCT for the ED visit has a probability value greater than 0. All regressions control for day of week using a series of indicator variables for Monday to Saturday, leaving Sunday as the reference day

Robust standard errors in parentheses

** p < 0.01, * p < 0.05

On the other hand, for encounters for which care is likely required, longer travel distances to the ED are associated with higher NYU ED scores. For EUA, each additional mile of travel distance to the ED is associated with increases in scores for almost all types of patients with different payment sources (Table 2). For the regressions related to *frequent* nonurgent ED use, we find that each additional mile in the *average* distances to the ED (because not all patients go to the same ED each time) is associated with fewer total numbers of NE, PCT, and PCP visits, but not for EUA visits (Table 3).

**Table 3:** Correlates of frequent ED utilization

	(1)	(2)	(3)	(4)
Variables	Log visits NE ≥ 0.75	Log visits PCT ≥ 0.75	Log visits PCP ≥ 0.75	Log visits EUA ≥ 0.75
Age				
≤1	0.00436	0.151**	0.0344**	−0.0668**
	(0.00488)	(0.00382)	(0.00804)	(0.00729)
1 – 17	0.0961**	0.0601**	0.107**	−0.00123
	(0.00413)	(0.00367)	(0.00704)	(0.00477)
18 – 34	0.260**	0.124**	0.148**	0.0933**
	(0.00388)	(0.00352)	(0.00682)	(0.00365)
35 – 64	0.216**	0.118**	0.127**	0.0658**
	(0.00364)	(0.00323)	(0.00588)	(0.00312)
Gender				
Female	0.101**	0.0317**	−0.0272**	−0.0152**
	(0.00146)	(0.00130)	(0.00299)	(0.00173)
Race/Ethnicity				
African American	0.00992**	0.0262**	0.135**	−0.0220**
	(0.00145)	(0.00133)	(0.00307)	(0.00196)
Asian	−0.147**	−0.0755**	−0.0311	−0.0570**
	(0.0119)	(0.0109)	(0.0262)	(0.0147)
Native American	−0.106**	−0.0547**	0.0431	−0.0423
	(0.0172)	(0.0149)	(0.0373)	(0.0240)
Hispanic	−0.139**	−0.0748**	−0.0133	−0.0705**
	(0.00437)	(0.00393)	(0.0106)	(0.00661)
Other race	−0.134**	−0.0741**	−0.0117	−0.0711**
	(0.00502)	(0.00463)	(0.0119)	(0.00689)
Expected source of payment				
Self-pay	0.0811**	0.0592**	0.0608**	0.0420**
	(0.00189)	(0.00183)	(0.00440)	(0.00247)
Medicare	0.126**	0.0984**	0.0790**	0.0341**
	(0.00321)	(0.00294)	(0.00565)	(0.00307)
Medicaid	0.162**	0.125**	0.112**	0.0697**
	(0.00211)	(0.00189)	(0.00436)	(0.00319)
HMO	−0.0187**	−0.00775*	0.00207	0.00717
	(0.00390)	(0.00378)	(0.00858)	(0.00427)
Other payer	−0.00316	−0.00810*	0.0168*	0.00362
	(0.00357)	(0.00356)	(0.00812)	(0.00419)
Average ED Distance	−0.000727**	−0.000781**	−0.000831**	2.68e-05
	(4.94e-05)	(4.89e-05)	(0.000108)	(5.11e-05)
Constant	−0.0140**	0.0421**	0.0673**	0.0789**
	(0.00400)	(0.00356)	(0.00684)	(0.00346)
Observations	539,376	441,688	121,310	176,652
R-squared	0.049	0.027	0.036	0.015

Standard errors in parentheses

** p < 0.01, * p < 0.05

We now return to the encounter-level analysis. Because FQHCs are intended primarily to serve vulnerable populations, we focus primarily on the association between greater travel distances to the FQHC and ED scores for self-pay, Medicare, and Medicaid patients. Our results show that for each additional mile further away that the closest FQHC is from the residence of Medicare patients, there is a greater likelihood of an NEPCT visit (0.0636, SE 0.0177). Conditional on a positive PCT score, each additional mile further away that the closest FQHC is from the residence of Medicare patients is also associated with a higher PCT score (0.052, SE 0.0183) (Table 2).

### Demographic characteristics and ED urgency scores

The overall pattern of the results demonstrates that younger patients are more likely to go to the ED for nonurgent medical conditions, and that the elderly are more likely to visit the ED for medical conditions for which emergency care is required. Female gender and all minorities show greater probabilities of visiting the ED for a condition with either a nonzero NE or PCT score. However, when we consider the probabilities for specific types of ED use (specifications 2 to 5, Table 2), a different pattern emerges. African Americans have higher NE and PCP scores, but lower EUA scores than White patients. On the other hand, Asians, Hispanics, and patients identifying as other race (s) have lower NE scores, but higher PCT scores.

Specifications focusing on frequent ED use show results broadly consistent with our encounter-level results above. Patients younger than 65 years generally have greater numbers of nonurgent ED visits than patients aged 65 years and older (Table 2). Female patients have more total ED visits considered nonurgent and fewer total ED visits likely to require emergency care. African Americans have more NE, PCT, and PCP visits during the 6-year period from 2005 to 2010. Self-pay, Medicare, and Medicaid patients have more ED visits of all types.

### Validity of the NYU ED Algorithm

We ran several regressions to assess the construct validity of the NYU ED Algorithm scores as applied to the South Carolina ED population. In Additional file 1: Table S1, we show that for all proxy measures of severity (inpatient admission within 30 days of an index ED visit, inpatient admission immediately upon the ED encounter, and total charges during the ED visit), the NYU ED Algorithm performed well. Nonurgent variables have consistently negative coefficients, indicating that the higher the score, the less likely the condition is urgent. On the other hand, urgent variables (PCP, EUA) have consistently positive coefficients, suggesting that higher scores are associated with more serious medical conditions.

## Discussion

Overall, our results are consistent with two primary interpretations. First, convenience (as measured by travel distances) appears to facilitate nonurgent use of the hospital ED for patients with nonpublic sources of insurance. Second, we find evidence of disparities in access to health care based on socioeconomic status, which seems to be alleviated somewhat by the proximity of an FQHC. These two findings are at odds with a common belief that Medicare and Medicaid patients abuse the system with low-acuity ED visits, a notion that underlies proposals to deny payment for nonurgent visits to the ED [20]. Evidence also suggests that nonurgent ED use may not be the cause of all negative consequences (such as ED overcrowding) commonly attributed to it [21-23]. Indeed, we find that in South Carolina, privately insured (rather than publicly insured) patients appear more likely to be influenced by convenience (as measured by travel distance). Vulnerable patients such as African Americans and Medicaid patients visit the ED more frequently and with higher NYU Algorithm scores for *both* nonurgent and urgent conditions, suggesting an unmet need in access to health care.

Indeed, health disparities potentially linked to socioeconomic status are detectable even from a visual inspection of the distribution of NE and PCT scores across zip codes in South Carolina (See Figs. 1 and 2). Relatively richer areas such as Georgetown, Charleston, and Pickens counties have some of the highest NE use in the state. However, the distribution of PCT patterns reflects county-level poverty measures better, particularly in Allendale, Bamberg, Marlboro, Dillon, and Marion counties, which are counties with some of the highest levels of poverty rates in South Carolina. This pattern casts doubt that the poor are the only ones who use the hospital ED for NE medical conditions. Rather, patients in poorer counties appear to use the ED for PCT reasons, which suggests that there are barriers to access to primary care among the state’s poor.

These results are borne out in our regressions estimates. Consistent with prior literature, patients with private, nonpublic sources of insurance coverage are the most likely to be influenced by travel distances when deciding to visit the ED [24]. Patients with nonpublic insurance appear to choose the ED when it is convenient because they are the only groups with lower acuity scores given greater travel distances to the ED. However, for other patients, we show that distances to the ED facility have no statistically significant relationship to nonurgent use scores. Greater travel distances are associated with higher emergent scores (PCP, EUA) for patients with almost all types of insurance coverage.

While our study is, to our knowledge, the first to investigate the associations between travel distances and measures of ED use based on *estimated urgency on a population level*, our frequency results echo the literature on travel convenience and *frequent* ED use (Table 2). In a retrospective analysis of a single level I trauma and tertiary care center in New England, Henneman et al. found that increasing travel distances were associated with decreasing ED visitation rates [25]. Another study found that Medicaid children who lived 1.19 miles further away from the ED had 11 % lower ED use than those who lived within 0.5 miles of the ED. The same study estimated that children who lived 3.13 miles or more away from their primary care physicians visited the ED 13 % more frequently than children who lived within 0.7 miles of their physicians [26]. Research has also shown that patients seek urgent care settings for their convenience, citing the ability to obtain same-day lab results and medications as important considerations [27]. In our analysis of South Carolina ED use patterns, convenience as a motivating factor appears to be much stronger for patients with private sources of payment, rather than Medicare or Medicaid patients (Table 2).

Our results present some evidence that a greater distance from safety net providers (FQHCs) is correlated with higher scores of nonurgent ED use for Medicare patients. These results are less evident than those associated with travel distances to the ED, but are consistent with a large body of literature linking more-frequent use of the ED to a lack of or barriers to access to a usual place of care [13, 14, 27-36]. A particularly relevant study showed that counties without primary care clinics at the community health center had 33 % higher all-cause ED visits than those with such clinics [37]. These barriers to primary care access come in many forms, including cultural incompatibility [38, 39], language barriers [40], lack of availability of timely care, financial considerations [27], and health illiteracy [41].

Our results also reveal patterns of ED use that suggest health disparities along racial and social lines, which is a recurrent theme in the literature on avoidable ED use [42]. In particular, African American and Medicaid patients have higher probabilities of using the ED for medical conditions with nonzero NE or PCT scores, as well as higher scores for NE and PCT medical reasons. Furthermore, African American and Medicare patients are the only two groups of patients in our analysis to visit the ED with higher PCP scores, which means that they visit the ED for medical conditions that could have been prevented with timely and appropriate ambulatory care. Because Medicare patients are elderly and likely require care for chronic medical conditions, the fact that African American patients seek medical treatment in the ED with higher PCP scores even when we control for age, gender, and expected source of payment may point to troubling health disparities in vulnerable populations in South Carolina.

These patterns are corroborated in our regressions using the number of ED visits over 6 years with NE, PCT, PCP, or EUA scores of 75 % or above. Medicare and Medicaid patients consistently have more visits for all reasons (NE, PCT, PCP, and EUA). In particular, African American patients have 13 % more ED visits that are at least 75 % likely to be PCP, even after controlling for age, gender, and insurance type. Numerous studies have confirmed that nationwide, African American and Medicaid patients in particular face barriers to primary care, use the ED more frequently [34, 43, 44] for more nonurgent medical reasons [35, 45, 46], and often use the hospital ED as their usual source of health care [47, 48].

That African American and Medicaid patients rely more heavily on the ED for routine medical treatment should concern healthcare policymakers. In some communities, these are the patients most at risk from hospital ED closures [49]. Even when they do receive care in the ED, African American patients have been shown to experience longer ED wait times for critical conditions such as stroke, other cerebrovascular conditions, or diabetes [6, 7]. Denying payments for nonurgent ED visits for Medicaid patients, as has been proposed, may exacerbate these existing barriers to health care [20].

## Conclusion

We show two overarching patterns in the urgency of ED use in South Carolina between 2005 and 2010. Convenience, as measured by distance to the ED facility, appears to have played a role in encouraging nonurgent ED utilization primarily among patients with private or no insurance coverage. Secondly, health care disparities exist, with African American and Medicaid patients showing more *frequent* use, and more *avoidable* use of the hospital ED. Moreover, African American patients seek ED care for conditions that are more likely to be PCP. This pattern of ED use points to possible barriers to primary care access for these vulnerable populations.

### Limitations

There are several limitations to this study. First, although we conducted basic construct validity tests, concerns remain that the NYU ED Algorithm does not provide accurate measures of medical urgency in the South Carolina population from 2005 to 2010. However, the NYU ED Algorithm is the most utilized measure to capture nonurgent use in administrative claims data, and it is difficult to conceive alternative methods to evaluate over 6.5 million individual ED visits. Also, for practical reasons, Billings et al. chose diagnostic codes assigned after the ED visit, rather than patients’ initial complaint [3]. It is possible that an initial fear of a heart attack may ultimately be diagnosed as esophageal reflux by the end of the visit [50]. Yet as used in our study, the Billings NYU ED Algorithm may provide useful information relevant to healthcare policymakers, particularly with respect to the nature and distribution of nonurgent ED use and barriers to access and subsequent health outcomes of vulnerable populations in South Carolina.

Second, although we were able to obtain the exact road distance between patients’ home address and FQHCs and ED facilities, there is no guarantee that patients actually traveled to the ED from home. Nevertheless, these distances are the best approximations for convenience of access in a population-based administrative claims database.

We note also that FQHC distance may not necessarily reflect the ease of access to alternative care. Underserved South Carolinians have other avenues for care, including other types of non-FQHC free clinics. Furthermore, distance to FQHCs alone may not reflect accessibility, as many underserved and traditionally underserved groups may not have access to private means of transportation and must rely on public transportation. FQHC proximity also does not necessarily guarantee access because of potential lags in appointment times and limited hours of operation. It is perhaps for these reasons that we fail to observe a greater impact of FQHC distance on measures of nonurgent ED use for Medicaid patients.

Finally, as for all observational studies, we cannot exclude the possibility of unmeasured confounding. By exploiting the temporal nature of the panel data structure for all but the frequent user regressions, and using the econometric technique of hospital and quarterly fixed effects, we obtain consistent estimates controlling for time-invariant confounding across hospitals and quarters. However, time-varying confounding sources of bias are not accounted for in our results.

## Additional file

Additional file 1:
**Validity of NYU ED measures.**

## Abbreviations

ED: Emergency Department
FQHC: Federally Qualified Health Center
HMO: Home Maintenance Organization
NE: Nonemergent
PCP: Primary care preventable
PCT: Primary care treatable
EUA: Emergent/Unavoidable

## References

[1] Weinick RM, Billings J, Thorpe J (2003). Ambulatory Care Sensitive Emergency Department Visits: A National Perspective. Abstr AcademyHealth Meet.

[2] Weber EJ, Showstack JA, Hunt KA, Colby DC, Grimes B, Bacchetti P (2008). Are the Uninsured Responsible for the Increase in Emergency Department Visits in the United States?. Ann Emerg Med.

[3] Nyu Ed Algorithm. Available at Http://Wagner.Nyu.Edu/Faculty/Billings/Nyued-Background.Php, Accessed August 15, 2014.

[4] Simon HK, Hirsh DA, Rogers AJ, Massey R, DeGuzman MA (2009). Pediatric Emergency Department Overcrowding: Electronic Medical Record for Identification of Frequent, Lower Acuity Visitors. Can We Effectively Identify Patients for Enhanced Resource Utilization?. J Emerg Med.

[5] Knapman M, Bonner A (2010). Overcrowding in Medium-Volume Emergency Departments: Effects of Aged Patients in Emergency Departments on Wait Times for Non-Emergent Triage-Level Patients. Int J Nurs Pract.

[6] Herring A, Wilper A, Himmelstein DU, Woolhandler S, Espinola JA, Brown DFM (2009). Increasing Length of Stay among Adult Visits to U.S. Emergency Departments, 2001–2005. Acad Emerg Med.

[7] Karve SJ, Balkrishnan R, Mohammad YM, Levine DA (2011). Racial/Ethnic Disparities in Emergency Department Waiting Time for Stroke Patients in the United States. J Stroke Cerebrovasc Dis.

[8] Fayyaz J, Khursheed M, Mir MU, Mehmood A (2013). Missing the Boat: Odds for the Patients Who Leave Ed without Being Seen. BMC Emerg Med.

[9] Aacharya R, Gastmans C, Denier Y (2011). Emergency Department Triage: An Ethical Analysis. BMC Emerg Med.

[10] Durand A-C, Gentile S, Devictor B, Palazzolo S, Vignally P, Gerbeaux P (2011). Ed Patients: How Nonurgent Are They? Systematic Review of the Emergency Medicine Literature. Am J Emerg Med.

[11] Johnson PJ, Ghildayal N, Ward AC, Westgard BC, Boland LL, Hokanson JS. Disparities in Potentially Avoidable Emergency Department (Ed) Care: Ed Visits for Ambulatory Care Sensitive Conditions. Med Care. 2012;50(12). 1020–1028 1010.1097/MLR.1020b1013e318270bad318274. 10.1097/MLR.0b013e318270bad4 23032354

[12] Oster A, Bindman AB (2003). Emergency Department Visits for Ambulatory Care Sensitive Conditions: Insights into Preventable Hospitalizations. Med Care.

[13] Lowe RA, Localio AR, Schwarz DF, Williams S, Tuton LW, Maroney S (2005). Association between Primary Care Practice Characteristics and Emergency Department Use in a Medicaid Managed Care Organization. Med Care.

[14] Kellermann AL, Weinick RM (2012). Emergency Departments, Medicaid Costs, and Access to Primary Care — Understanding the Link. N Engl J Med.

[15] Uscher-Pines L, Pines J, Kellermann A, Gillen E, Mehrotra A (2013). Emergency Department Visits for Nonurgent Conditions: Systematic Literature Review. Am J Manag Care.

[16] HRSA Data Warehouse Health Care Service Delivery and Look-Alike Sites Data Download, available at http://datawarehouse.hrsa.gov/data/dataDownload/hccDownload.aspx, last accessed March 26, 2015.

[17] Hausman JA, Taylor WE (1981). Panel data and unobservable individual effects. Econometrica: Journal of the Econometric Society.

[18] Papke LE, Wooldridge JM (2008). Panel Data Methods for Fractional Response Variables with an Application to Test Pass Rates. J Econ.

[19] Profile of General Demographic Characteristics, Available at Http://Factfinder2.Census.Gov/Faces/Tableservices/Jsf/Pages/Productview.Xhtml?Src=Bkmk, Accessed December 14, 2014

[20] Adams JG (2013). Emergency Department Overuse: Perceptions and Solutions. JAMA.

[21] Pitts SR, Pines JM, Handrigan MT, Kellermann AL (2012). National Trends in Emergency Department Occupancy, 2001 to 2008: Effect of Inpatient Admissions Versus Emergency Department Practice Intensity. Ann Emerg Med.

[22] United States General Accounting Office: Hospital Emergency Departments: Crowde Conditions Vary among Hospitals and Communities. In*.*, vol. GAO-03-460, available at http://www.gao.gov/new.items/d03460.pdf; 2003.

[23] United States General Accounting Office: Hospital Emergency Departments: Crowding Continues to Occur, and Some Patients Wait Longer Than Recommended Time Frames. In*.*, vol. GAO-09-347, available at http://www.gao.gov/assets/290/289048.pdf; 2009.

[24] Rubin MA, Bonnin MJ (1995). Utilization of the Emergency Department by Patients with Minor Complaints. J Emerg Med.

[25] Henneman PL, Garb JL, Capraro GA, Li H, Smithline HA, Wait RB (2011). Geography and Travel Distance Impact Emergency Department Visits. J Emerg Med.

[26] Ludwick A, Fu R, Warden C, Lowe RA (2009). Distances to Emergency Department and to Primary Care Provider’s Office Affect Emergency Department Use in Children. Acad Emerg Med.

[27] Scott D, Batal H, Majeres S, Adams J, Dale R, Mehler P (2009). Access and Care Issues in Urban Urgent Care Clinic Patients. BMC Health Serv Res.

[28] Tranquada KE, Denninghoff KR, King ME, Davis SM, Rosen P (2010). Emergency Department Workload Increase: Dependence on Primary Care?. J Emerg Med.

[29] Burns W (2010). Emergency Department Utilization among the Poor: The Need to Improve Access to Quality Primary Care. Retrieved February.

[30] Christakis DA, Mell L, Koepsell TD, Zimmerman FJ, Connell FA (2001). Association of Lower Continuity of Care with Greater Risk of Emergency Department Use and Hospitalization in Children. Pediatrics.

[31] Gresenz CR, Rogowski J, Escarce JJ (2007). Health Care Markets, the Safety Net, and Utilization of Care among the Uninsured. Health Serv Res.

[32] Hossain MM, Laditka JN (2011). The Influence of Rurality on the Volume of Non-Urgent Emergency Department Visits. Spat Spatiotemporal Epidemiol.

[33] Mathison DJ, Chamberlain JM, Cowan NM, Engstrom RN, Fu LY, Shoo A (2013). Primary care spatial density and nonurgent Emergency Department Utilization: a new methodology for evaluating access to care. Acad Pediatr.

[34] Sandoval E, Smith S, Walter J, Schuman S-AH, Olson MP, Striefler R (2010). A Comparison of Frequent and Infrequent Visitors to an Urban Emergency Department. J Emerg Med.

[35] Sharma V, Simon SD, Bakewell JM, Ellerbeck EF, Fox MH, Wallace DD (2000). Factors Influencing Infant Visits to Emergency Departments. Pediatrics.

[36] Sturm JJ, Hirsh DA, Lee EK, Massey R, Weselman B, Simon HK (2010). Practice Characteristics That Influence Nonurgent Pediatric Emergency Department Utilization. Acad Pediatr.

[37] Rust G, Baltrus P, Ye J, Daniels E, Quarshie A, Boumbulian P (2009). Presence of a Community Health Center and Uninsured Emergency Department Visit Rates in Rural Counties. J Rural Health.

[38] Vaughn LM, Jacquez F (2012). Characteristics of Newly Immigrated, Spanish-Speaking Latinos Who Use the Pediatric Emergency Department: Preliminary Findings in a Secondary Migration City. Pediatr Emerg Care.

[39] Wilkin HA, Cohen EL, Tannebaum MA (2012). How low-income residents decide between emergency and primary health care for non-urgent treatment. Howard Journal of Communications.

[40] Downey LVA (2010). Rate of Use of Referrals in the Emergency Department. J Health Manag.

[41] Morrison AK, Myrvik MP, Brousseau DC, Hoffmann RG, Stanley RM (2013). The relationship between parent health literacy and pediatric Emergency Department Utilization: a systematic review. Acad Pediatr.

[42] Khan Y, Glazier RH, Moineddin R, Schull MJ (2011). A Population-Based Study of the Association between Socioeconomic Status and Emergency Department Utilization in Ontario, Canada. Acad Emerg Med.

[43] Wajnberg A, Hwang U, Torres L, Yang S (2012). Characteristics of Frequent Geriatric Users of an Urban Emergency Department. J Emerg Med.

[44] Timm NL, Ruddy RM (2010). Demographics of Patient Visits During High Daily Census in a Pediatric Ed. Am J Emerg Med.

[45] Chiou S-J, Campbell C, Myers L, Culbertson R, Horswell R (2010). Factors Influencing Inappropriate Use of Ed Visits among Type 2 Diabetics in an Evidence-Based Management Programme. J Eval Clin Pract.

[46] Buesching DP, Jablonowski A, Vesta E, Dilts W, Runge C, Lund J (1985). Inappropriate Emergency Department Visits. Ann Emerg Med.

[47] Phillips K (2010). Ps3-22: Medical Homes: a comparison of the factors that predict utilization across the lifespan. Clin Med Res.

[48] Tang N, Stein J, Hsia RY, Maselli JH, Gonzales R (2010). Trends and Characteristics of Us Emergency Department Visits, 1997–2007. JAMA.

[49] Hsia RY, Srebotnjak T, Kanzaria HK, McCulloch C, Auerbach AD (2012). System-Level Health Disparities in California Emergency Departments: Minorities and Medicaid Patients Are at Higher Risk of Losing Their Emergency Departments. Ann Emerg Med.

[50] Raven MC, Lowe RA, Maselli J, Hsia RY (2013). Comparison of Presenting Complaint Vs Discharge Diagnosis for Identifying “Nonemergency” Emergency Department Visits. JAMA.

