# Supplementary material for: Travel distances, socioeconomic characteristics, and health disparities in nonurgent and frequent use of Hospital Emergency Departments in South Carolina: a population-based observational study
Source: BMC Health Serv Res. 2015 May 16;15:203. doi: 10.1186/s12913-015-0864-6 (PMC4448557; doi:10.1186/s12913-015-0864-6)
Supplement: Additional file 1: — Validity of NYU ED measures. [file 12913_2015_864_MOESM1_ESM.doc]

| **Additional file 1 Validity of NYU ED measures.** | | | |
| --- | --- | --- | --- |
|  |  |  |  |
|  | **(1)** | **(2)** | **(3)** |
| **Variables** | **Refer to Inpatients** | **Inpatient within 30 days** | **Log Charges** |
|  |  |  |  |
| **NYU ED variables** |  |  |  |
| Nonemergent | -0.0118** | -0.00580** | -0.409** |
|  | (0.00377) | (0.00179) | (0.0200) |
| Primary care treatable | -0.0357** | -0.0114** | -0.154** |
|  | (0.00313) | (0.00174) | (0.0285) |
| Primary care preventable | 0.0149* | 0.0625** | 0.536** |
|  | (0.00708) | (0.00316) | (0.0166) |
| Emergent/Unavoidable | 0.0739** | 0.0673** | 1.100** |
|  | (0.00880) | (0.00843) | (0.0451) |
| **Age** |  |  |  |
| ≤ 1 | -0.0213** | -0.0564** | -0.918** |
|  | (0.00414) | (0.00183) | (0.0313) |
| 1 –17 | -0.0263** | -0.0625** | -0.717** |
|  | (0.00316) | (0.00196) | (0.0202) |
| 18 – 34 | -0.0200** | -0.0460** | -0.396** |
|  | (0.00338) | (0.00341) | (0.0140) |
| 35 – 64 | -0.0160** | -0.0304** | -0.212** |
|  | (0.00292) | (0.00194) | (0.00918) |
| **Gender** |  |  |  |
| Female | -0.00661** | -0.00131 | 0.0212** |
|  | (0.000697) | (0.00153) | (0.00523) |
| **Race/Ethnicity** |  |  |  |
| African American | -0.00440** | -0.00591** | -0.141** |
|  | (0.000737) | (0.000495) | (0.00822) |
| Asian | -0.00378 | -0.00297 | -0.0162 |
|  | (0.00207) | (0.00407) | (0.00926) |
| Native American | 0.00306 | -0.00794** | -0.0873 |
|  | (0.0121) | (0.00169) | (0.0465) |
| Hispanic | -0.00820* | -0.00492** | -0.0471** |
|  | (0.00333) | (0.00119) | (0.00930) |
| Other race | -0.00366 | -0.00198 | -0.0284* |
|  | (0.00255) | (0.00237) | (0.0139) |
| **Expected source of payment** |  |  |  |
| Self-pay | 0.0128** | -0.0129** | -0.112** |
|  | (0.00274) | (0.00108) | (0.0219) |
| Medicare | 0.0203** | 0.0294** | -0.00176 |
|  | (0.00373) | (0.00189) | (0.0156) |
| Medicaid | 0.00962** | 0.00847** | -0.132** |
|  | (0.00184) | (0.00102) | (0.0188) |
| HMO | -0.00106 | 0.000790 | 0.0131 |
|  | (0.000929) | (0.000514) | (0.00712) |
| Other payer | 0.00646** | 0.000434 | -0.0415** |
|  | (0.00203) | (0.00221) | (0.0155) |
|  |  |  |  |
| Observations | 6592501 | 6592501 | 6589950 |
| R-squared | 0.016 | 0.030 | 0.234 |
| Number of hospitals | 64 | 64 | 64 |
|  |  |  |  |
|  |  |  |  |

Robust standard errors in parentheses

** p<0.01, * p<0.05
